# T cell exhaustion: from pathophysiological basics to tumor immunotherapy

**DOI:** 10.1186/s12964-016-0160-z

**Published:** 2017-01-05

**Authors:** Kemal Catakovic, Eckhard Klieser, Daniel Neureiter, Roland Geisberger

**Affiliations:** 1 Laboratory for Immunological and Molecular Cancer Research, Department of Internal Medicine III with Haematology, Medical Oncology, Haemostaseology, Infectiology and Rheumatology, Oncologic Center, Paracelsus Medical University, Müllner Hauptstrasse 48, Salzburg, 5020 Austria; 2 Salzburg Cancer Research Institute, Salzburg, Austria; 3 Department of Pathology, Paracelsus Medical University, Müllner Hauptstrasse 48, Salzburg, 5020 Austria

**Keywords:** Immunotherapy, PD-1, PD-L1, T cell exhaustion, Cancer

## Abstract

The immune system is capable of distinguishing between danger- and non-danger signals, thus inducing either an appropriate immune response against pathogens and cancer or inducing self-tolerance to avoid autoimmunity and immunopathology. One of the mechanisms that have evolved to prevent destruction by the immune system, is to functionally silence effector T cells, termed T cell exhaustion, which is also exploited by viruses and cancers for immune escape In this review, we discuss some of the phenotypic markers associated with T cell exhaustion and we summarize current strategies to reinvigorate exhausted T cells by blocking these surface marker using monoclonal antibodies.

## Background

Exhausted T cells can be distinguished from other T cell dysfunctions such as anergy and senescence based on their underlying molecular mechanisms [1]. Whereas anergy is introduced during priming due to the absence of costimulatory signals and senescence is growth arrest after extensive proliferation [2] exhausted T cells arise from cells, which initially gained effector function, but become gradually silenced due to continous T cell receptor (TCR) stimulation from persistent antigen [3].

T cell exhaustion has been initially observed in mice infected with the lymphocytic choriomeninigits virus (LCMV), where a chronically persistent virus strain rendered virus specific cytotoxic T cells non-functional. Using the same mouse model, reversibility of T cell exhaustion could be demonstrated [4, 5].

**Fig. 1:**
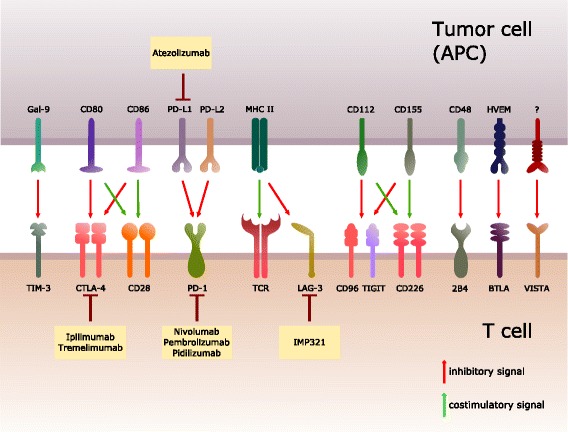
Inhibitory/costimulatory receptors and their corresponding ligands. Schematic overview of inhibitory/ costimulatory receptors expressed by T cells interacting with their counterpart on antigen-presenting cells (APCs) or tumor cells. Additionally, various blocking antibodies against inhibitory receptors or their ligands in clinical trials are depicted with the aim of reversing T cell exhaustion

Exhausted T cells have also been observed in response to several other virus infections like simian immunodeficiency virus (SIV), human immunodeficiency virus (HIV), hepatitis B virus (HBV), hepatitis C virus (HCV) and human T lymphotropic virus 1 (HTLV1) [6–15]. However, mice with impeded T cell exhaustion develop severe spontaneous autoimmune diseases and succumb to fatal CD8 T cell-mediated immune pathologies during early systemic LCMV infection, showing that T cell exhaustion substantially contributes to peripheral tolerance and to moderate immune responses [16, 17]. In line with that, presence of exhausted T cells in patients with autoimmune diseases correlates with favorable prognosis [18]. T cell exhaustion has also been observed in tumor patients, where the exhaustion of tumor specific T cells is suggested to impede clearance of the tumor, thus contributing to tumor immune escape [19–23]. Characteristics of exhaustion are are continuous enhancement of T cell dysfunction due to persistent antigen exposure, an increased expression of multiple inhibitory receptors (IR), theprogressive loss of effector cytokine secretion (IL-2, Interferone gamma [IFNγ], Tumor necrosis factor alpha [TNFα]), analtered cell metabolism and a markedly different transcriptional profile [20, 21, 23–26]. The gradual dysfunction of exhausted T cells is accompanied by the expression of IRs, which wire inhibitory signals to the nucleus upon interaction with ligands on target cells (Fig. 1 and Table 1). However, recent reports reveal that T cells do not uniformly exhaust during chronic diseases or cancer, but that specific subsets with different memory-like or proliferative potentials emerge upon exposure to persisting anigen [27–29]. As blocking iR/ligand interactions (so called immune checkpoint inhibition) seems an appealing strategy to partially reverse T cell exhaustion and to possibly regain anti-cancer immunity, a set of most promising inhibitory receptors (although their expression is not exclusively restricted to exhausted T cells) and current approaches to impede their function in context of current cancer therapies are discussed in this review:

**Table 1:** Expression, ligands and signaling pathways of immune checkpoint molecules (based on [210] and [211])

Immune checkpoint receptors (*synonym*)	Cellular expression	Ligand	Intracellular motif	Signaling pathways
CTLA-4 (*CD152*)	T cells	CD80, CD86	YxxM	SHP2, LCK/ZAP70/PI3K PP2A/AKT
PD-1 (*CD279*)	T cells, B cells, DCs, NKT cells, Monocytes	PD-L1, PD-L2	ITIM, ITSM	SHP1, PI3K/AKT SHP2, LCK/ZAP70/PI3K, RAS
TIGIT (*VSIG9, VSTM3*)	T cells, NK and NKT cells	CD155, CD112	2 × ITIM	NF-kB, PI3K and MAPK
LAG-3 (*CD223*)	T cells, B cells, DC, NK cells	MHCII	KIEELE	not determined
2B4 (*CD244*)	T cells, NK cells, Monocytes, Basophiles	CD2, CD48	ITSM	not determined
BTLA (*CD272*)	T cells, B cells, DC, Macrophages, Myeloid cells	HVEM, CD80	ITIM, ITSM	SHP1, PI3K/AKT SHP2, LCK/ZAP70/PI3K
TIM3 (HAVCR2)	T cells, B cells, NK cells, NKT cells, DCs, Macrophages	Gal-9	Y235, Y242	PI3K BAT3/LCK
VISTA (PD1-H)	T cells, DCs, Macrophages, Monocytes, Neutrophils	not determined	not determined	not determined
CD96 (Tactile)	T cells, NK cells, Myeloid cells	CD155	ITIM	not determined

## Inhibitory receptors associated with T cell exhaustion

### Cytotoxic T-lymphocyte-associated Protein 4 (CTLA-4)

CTLA-4 counteracts the positive signal mediated by CD28 by competing for the same ligands (CD80/86) with higher affinity [30–32]. CTLA-4 transmits signals by intracellularily binding the phosphatases PP2A and SHP-2. In addition, CTLA-4 is able to entrap its ligands CD80/CD86 by trans-endocytosis followed by degradation [33, 34].

CTLA-4 is up-regulated upon activation on naïve T cells and constitutively expressed on regulatory T cells (Tregs), since CTLA-4 is a transcriptional target of Foxp3, a key transcriptional factor of this subset [35, 36]. The role of CTLA-4 in immune suppression and tolerance has been validated in autoimmune mouse models such as type I diabetes and multiple sclerosis, where CTLA-4 blockade results in increased severity of the inflammatory phenotype [37]. CTLA-4 knockout mice provide additional evidence for its role as negative regulator of the immune response, due to the enhanced lymphoproliferative disorder and multiorgan tissue destruction [38, 39]. Paradoxically, although CTLA-4 decreases effector functions of CD4^+^ and CD8^+^ T cells, it increases the suppressive capacity of Tregs. For example, specific CTLA-4 knockdown or blockade on Tregs results in T cell mediated autoimmune disease and contributes to antitumor immunity. Additionally, CTLA-4 expressing Tregs mediate the downregulation of CD80/CD86 on antigen presenting cells and thereby reduce activation of naïve T cells [40, 41]. In context of cancer, it is suggested that CTLA-4 expression on low-affinity tumor specific T cells attenuates their proliferation which could be possibly overcome by CTLA-4 blockade. In addition, CTLA-4 expression on tumor specific Tregs could contribute to tumor immune escape by increasing the suppressive anti-tumor immunity and by downregulating CD80/CD86 on antigen presenting cells [42].

Thus, CTLA-4 dampens T cell activation, decreases the efficacy of antigen presenting cells to activate T cells and augments Treg mediated immune suppression.

### Programmed cell death 1 (PD-1)

Whereas CTLA-4 predominantly regulates initial T cell activation, the inhibitory receptor programmed cell death 1 (PD-1) is dampening effector T cell functions [43, 44]. Transient PD-1 cell surface expression is initiated upon T cell activation, but sustained expression is a characteristic marker of T cell exhaustion [45]. However, recent data show that PD-1 is not required for initiating T cell exhaustion and that absence of PD-1 even promotes accumulation of exhausted CD8^+^ T cells in mice [46]. The intracellular domain consists of an immunoreceptor tyrosine- based inhibitory motif (ITIM) and an immunoreceptor tyrosine- based switch motif (ITSM). PD-1 engagement with its ligand (PD-L1 or PD-L2) results in ITIM/ ITSM phosphorylation and subsequent recruitment of the phosphatases SHP1/ SHP2, which negatively regulate PI3K/ AKT and RAS signaling pathways [47–49]. In addition to CTLA-4 Tregs also express PD-1 on their cell surface [50]. During chronic infections such as LCMV, two subsets of exhausted T cells have been identified according to their transcriptional profile and expression of the inhibitory receptor PD-1 [51].

T cells with an increase in the transcription factor T-bet and an intermediate expression of PD-1 (T-bet^high^ PD-1^int^) retain residual secretion of IFNγ, TNFα and a limited proliferation rate. On the contrary, high levels of Eomesodermin (Eomes) and PD-1 (Eomes^high^ PD-1^high^) exhibited higher Blimp1and granzyme B production, co-expression of additional inhibitory receptors (CD160, Lag-3, 2B4, Tim-3) and are associated with a severe state of exhaustion, despite of a greater cytotoxic activity compared to T-bet^high^ PD-1^int^ T cells. Additionally, T-bet^high^ PD-1^int^ give rise to Eomes^high^ PD-1^high^ in an antigen driven manner and therefore count as a progenitor subset [51]. However, opposing data show that during chronic infection, a small subset of CD8^+^ T cells which were T cel factor 1 (Tcf1)^+^, PD-1^+^ and Eomes^+^ sustained a memory-like T cell response [28].

The blockade of the PD-1/PD-L1 axes in chronic infected LCMV mice sufficiently induces an antiviral state, by which two subpopulations of CD8 cells were identified. Whereas Eomes^high^ PD-1^high^ T cells exhibit a poor response to PD-1 pathway blockade, T-bet^high^ PD-1^int^ virus specific CD8 T cells efficiently reverse exhaustion and induce protective immunity in vivo suggesting that only a small fraction of exhausted T cells might overcome exhaustion by blocking PD-1 signaling [52].

### T cell immunoreceptor with Ig and ITIM domains (TIGIT)

Genome wide search for genes specifically expressed on immune cells and consisting of an extracellular Ig domain, type I transmembrane region together with either ITIMs or immunoreceptor tyrosine-based activation motifs (ITAMs), have revealed the existence of an additional inhibitory receptor namely T cell immunoreceptor with Ig and ITIM domains (TIGIT) [53, 54]. It belongs to the type 1 transmembrane proteins with an cytoplasmatic tail containing an immunoglobulin tail tyrosine (ITT)- like phosphorylation motif and ITIM [55]. Its expression is widely distributed across various T cell subsets including follicular helper T cells (T_FH_), Tregs, activated/memory T cells, natural killer (NK) and natural killer T (NKT) cells [53, 54, 56]. TIGIT attachment to poliovirus receptors (PVR) CD155/ CD112 results in the Grb2 mediated- recruitment of the SHIP1 phosphatase and downstream inhibition of NF-kB, PI3K and MAPK pathways [57, 58]. PVRs are expressed on APCs, endothelial cells, epithelial cells, but also on a number of tumor cells, which are inducible by Ras activation, Toll-like receptor (TLR) engagement and genotoxic stress [59–64].

Similar to CTLA-4/CD28 interactions, TIGIT shares the same ligands as the costimulatory molecule CD226 and competes for ligation resulting in the inhibition of T cell activation [65]. Interestingly, TIGIT is also capable of directly preventing the homodimerization of CD226 [65] leading to impaired TIGIT/CD226 balance, which impedes CD8 and NK cell antitumor and antiviral T cell response [66, 67]. Additionally, experiments in CD226 deficient mice showed impaired T cell proliferation, reduced immunological synapse formation and antitumor cytotoxicity [68]. Whereas an agonistic TIGIT antibody decreases T cell activation via CD3/CD28 stimulation, TIGIT knockdown enhances T cell proliferation, effector cytokine production such as IFNγ, IL-2 while decreasing IL-10 levels [69]. Additionally, circulating TIGIT^+^ T_FH_ cells produce higher levels of IL-21 and IL-4 and decreased IFNγ secretion compared to TIGIT^−^ T_FH_ cells promoting the differentiation and activation of B cells upon chronic stimulation [56]. Notably, the transcription factor FoxP3 regulates TIGIT expression and furthermore TIGIT^+^ Tregs exhibit higher suppressive functions compared to TIGIT^−^ Tregs [70, 71]. Besides the expression of additional inhibitory receptors, TIGIT^+^ Tregs are promoting Th2 responses by attenuating the secretion of the pro-inflammatory cytokines IFNγ and IL-17 [71].

Pre-clinical tumor studies showed that the specific co-inhibition of the TIGIT and PD-1 checkpoint axis causes a significant enhancement of anti-melanoma immune responses by increasing the effector function of cytotoxic T cells [72, 73]. Additionally, TIGIT positive tumor infiltrating CD8 T-cells could be detected in other solid-tumor entities such as small-cell lung carcinomas and colorectal carcinomas [65, 74]. Taken together, the combination of an anti-TIGIT and anti-PD-1 therapy could be a promising approach with associated stratified tumor entities in the future.

### Lymphocyte-activated gene-3 (LAG-3)

The cell surface protein lymphocyte-activated gene-3 (LAG-3) shows structural homologies to CD4 and binds MHCII with a higher affinity compared to CD4 [75, 76]. LAG-3 was also shown to interact with LSECTin, a surface lectin of the DC-SIGN family which is expressed on dendritic cells and also on tumor tissue [77]. LAG-3 is expressed on various cells such as B-cells, NK-cells, plasmacytoid dentritic cells, activated CD4, Tregs and CD8 T cells [78–81]. In the case of T cells, LAG-3 is transiently expressed upon activation and becomes internalized and degraded in the lysosomal compartments [82]. On the cell surface, LAG-3 co-distributes with TCR-CD3, binds to MHCII and inhibits CD4-dependent downstream signaling via its cytoplasmatic KIEELE motif and interestingly, not by disrupting CD4- MHCII engagement [83, 84]. As a result, LAG-3 exhibits a negative impact on T cell activation and effector function in vivo and vitro. Upon LAG-3 blockade in vitro T cell proliferation and cytokine production (mainly Th1 cytokines) increases and LAG-3 deficient T cells generate a larger pool of memory cells due to a delayed cell cycle arrest [85, 86]. An additional subtype of Tregs has been described coexisting in parallel to the classical CD4^+^Foxp3^+^ Treg cells called type 1 regulatory T cells (Tr1), which are lacking the expression of the transcription factor Foxp3 [87]. Tr1 cells exhibit immunosuppressive functions such as IL-10 and TGF-β secretion, however, LAG-3 blockade results in decreased suppressive activity in vivo and *vitro* pointing out a role for LAG-3 in Treg induction and expansion [88]. Similar to other exhaustion markers, LAG-3 is up-regulated in cancer and chronic infections. During chronic LCMV infections in mouse models combinatorial blockade of PD-1 and LAG-3 initiates synergistic control of viral load and improves T cell response in vivo [89]. Also various human cancer entities as well as tumor mouse models exhibit co-expression of PD-1 and LAG-3 on tumor-infiltrating T cells (TILs) [90, 91]. Interestingly, single inhibition of either LAG-3 or PD-1 alone does not result in improved control of chronic infection or tumor growth, pointing out the complex interactions among inhibitory receptors, whereby dual blockade synergistically reverses the exhausted phenotype [89, 91].

### 2B4

The receptor 2B4 (CD244) belongs to the signaling lymphocyte activation molecule (SLAM) subfamily within the immunoglobulin superfamily (IgSV). All members of this family contain two or more immunoreceptor tyrosine-based switch motifs (ITSMs) in their cytoplasmatic tail including the receptors CD229, CS1, NTB-A and CD84 [92]. 2B4 is expressed by NK cells, γδ T cells basophils and monocytes, upon activation on CD8^+^ T cells and binds with high affinity to CD48 on lymphoid and myeloid cells [93–95]. An additional binding partner of CD48 is CD2, which is suggested to contribute to the formation of lipid rafts and provides costimulatory signals [96]. Similar to the situation of TIGIT, 2B4- CD48 interaction exhibits either direct intracellular signaling or disruption of CD2-CD48 engagement. Interestingly, 2B4 is not a simple inhibitory receptor, indeed it can also exert costimulatory functions, depending on various factors. For example, 2B4 expression level, usage of downstream adaptor proteins (SAP or EAT-2) and it depends also on which of the four ITSMs is posphorylated [97–99].

2B4 is associated with T cell exhaustion. Various studies revealed, that exhausted CD8^+^ T cells exhibit increased 2B4 expression during chronic human diseases such as LCMV, HBV, HCV, HIV and also melanoma [100–105]. Interestingly, the adaptor protein SAP contributes to a positive 2B4 signaling, which is higher expressed in effector T cells compared to exhausted T cells, whereas the exhausted ones display elevated 2B4 levels in chronic LCMV infection [100, 106]. This leads to the suggestion, that the SAP/2B4 ratio is decreased, contributing to the T cell dysfunction during chronic antigen exposure.

### B and T lymphocyte attenuator (BTLA)

The cell surface protein B and T lymphocyte attenuator (BTLA) shares structural similarities with PD-1 and CTLA-4 and is expressed on T cells, B cells, macrophages and mature dentritic cells (DC) [107, 108]. Just like LAG-3, BTLA is transiently up-regulated upon TCR engagement and down-regulated on fully activated T cells, albeit retaining PD-1 and CTLA-4 expression [108]. Interestingly, only Th1 polarized cells maintain BTLA cell surface expression but not Th2 cells [107, 108]. The herpesvirus entry mediator (HVEM), which is expressed on various cell types (DCs, NK cells, T and B cells), binds to BTLA and also to the inhibitory receptor CD160 and the costimulatory receptor LIGHT [109, 110]. BTLA- HVEM engagement in T cells leads to tyrosine phosporylation on the conserved intracellular ITIM, inducing recruitment of the Src homology domain 2 (SH2)-containing protein tyrosine phosphatases SHP-1 and SHP-2 resulting in diminished CD3-induced secretion of IL-2 and T cell proliferation [108, 111].

Since BTLA is described as an inhibitory receptor, it is associated with peripheral tolerance. BTLA deficient mice develop autoimmune hepatitis- like disease with elevated levels of self antibodies, activated CD4^+^ T cells in the periphery, inflammatory cell infiltration of various organs and reduced survival [112]. Similar results have been achieved by the usage of BTLA-deficient T cells exhibiting increased susceptibility to experimental autoimmune encephalomyelitis EAE [108]. Interestingly, a single administration of agonistic BTLA antibodies at the time of autologous haematopoietic stem cell transplantation prevents the development of graft- versus- host disease by the inhibition of CD4^+^ Foxp3^−^ effector T cell expansion [113]. Furthermore, agonistic BTLA antibodies prolong murine cardiac allograft survival by decreasing IL-2 and IFNγ production and shifting the differentiation towards the Treg phenotype [114]. Additionally to the function as receptor, BTLA can also behave as ligand. This have been proved by several studies, indicating that HVEM elicits pro- survival signal for effector and memory T cells expressing HVEM [115–117].

Overexpression in human cancer [118], especially in hematological tumors [119], is linked to impaired tumor specific T-cell activity [23, 120]. Focusing on malignant melanoma, the triple blockade of PD1, TIM3 and BTLA leads consecutively to an increased expansion, proliferation and cytokine production of tumor-associated antigen- specific CD8^+^ T-cells [121]. Comparably to malignant melanoma, a heterogeneous amount of PD-1, Tim-3, CTLA-4, LAG-3, and BTLA were expressed on intratumoral CD8^+^ T cells from 32 patients with NSCLC. Furthermore, these findings could be linked to progression of the disease [122]. Interestingly, this investigation could clearly demonstrate, that the expression of these immune checkpoint inhibitors was time-dependent showing an early PD-1 and late LAG-3/BTLA expression [122]. Another study with NSCLS could relate the expression of PD-L1, PD-L2, PD-1, TIM-3, B7-H3, BTLA and CTLA-4 to the carcinogenesis relevant epithelial-mesenchymal transition [123]. In another animal model, investigating thyroid carcinoma, a combination of vaccination with BTLA inhibition lead to tumor regression [124]. Furthermore, it was shown that BTLA plays a role in suppression of tumor-associated antigen-specific CD8^+^ T-cell kind allogeneic stem-cell transplantation [125].

### T-cell immunoglobulin and mucin- containing protein 3 (TIM3)

The inhibitory receptor T-cell immunoglobulin and mucin- containing protein 3 (TIM-3) is regulated by the transcription factor T-bet and expressed on various T cell subsets including Th1, CD8^+^, Tregs but also on DCs, macrophages and monocytes [126, 127]. Although TIM-3 is thought to exhibit suppressive functions it does not contain an ITIM motif in its intracellular domain like PD-1 or TIGIT. It binds to the soluble molecule S-type lectin Galectin-9 (Gal-9), which is upregulated by IFNγ leading to the downstream recruitment of the Src family tyrosine kinase Fyn and the p85 phosphatidylinositol 3-kinase (PI3K) adaptor [128, 129]. As a result, Th1 mediated immunity is impaired by reducing IFNγ production, increased apoptosis in Th1 and cytotoxic CD8^+^ T cell in vitro [130, 131]. Other ligands for TIM3 are carcinoembryonic antigen cell adhesion molecule 1 (CEACAM1) [132], HMGB1 [133] and phosphatidylserine [134]. In preclinical studies, it could be shown that, blockade of TIM-3 signaling enhances the skewing from Th2 to Th1 subsets, thereby reducing allergen induced airway inflammation. Inhibition of Gal-9 amplifies symptoms of experimental autoimmune encephalomyelitis acute graft-versus host disease and type I diabetes in non-obese (NOD) mice [135–138]. The role of TIM-3 is currently being controversially discussed. Some studies display a negative impact on Th1 and Th17 polarization in vitro, while others suppose that Gal-9 triggers Treg differentiation or inhibits Th17 skewing in a TIM-3 independent manner [139–142]. Antagonistic TIM-3 antibodies increases the secretion of Th1 and Th17 effector cytokine production in vitro, elevated Th1 and Th17 differentiation in vivo and diminishes Treg conversion in vitro and in vivo [138, 143, 144]. TIM-3 expression on CD8^+^T cells is associated with high degree of dysfunction in various chronic infections, but also in lymphoma and melanoma patients [145–148]. As discussed in the last section, antagonizing TIM-3 signaling contributes to tumor regression and control of viral load, which can be potentiated by additional PD-1 blockade [146, 149–151].

### V domain Ig suppressor of T cells activation (VISTA)

Cloning of a Treg specific transcript with homology to the Ig superfamily led to the discovery of the V domain Ig suppressor of T cells activation (VISTA) or also known as PD-1 homolog (PD-1H) [152, 153]. This type I transmembrane protein consists of 7 exons and shares 85,6% similarity between human and mouse [153]. Although it is suggested that VISTA shares homology with either PD-1 or PD-L1, it does not contain ITIMs or ITAMs [152, 154]. However, due to the fact that the cytoplasmatic tail contains two protein kinase C binding sites and proline residues, which potentially function as docking sites, VISTA may act as both receptor and ligand such as the inhibitory receptor BTLA [154]. Interestingly, the binding partner of VISTA is still unknown. VISTA expression is not limited to T cells. Indeed, is also expressed by DCs, macrophages, monocytes and neutrophils [152, 153, 155]. Besides CTLA-4, PD-1 and TIGIT, Tregs additionally express VISTA on their cell surface, which is suggested to contribute to Treg differentiation and to their suppressive function. Several studies offer solid evidence for VISTAs immunomodulatory role. Firstly, VISTA-fusion protein promotes Treg differentiation in vitro [155]. Secondly, blockade of VISTA impairs differentiation of tumor-specific Tregs, whereby decreasing Treg-mediated suppression and increases infiltration, proliferation and effector functions of tumor-specific T cells [156]. The role of VISTA as a negative regulator of T cell mediated immune response has been strengthened by the fact that VISTA deficient mice display elevated T cell activation, proliferation, secretion of inflammatory cytokines (IFNγ, TNFα, monocyte chemotactic protein-1 [MCP-1], IL-6), chemokines (interferone gamma induced protein-10 [IP-10], monocyte interferon gamma inducing factor [MIG], MCP-1) and multiorgan chronic inflammation. This inflammatory phenotype is synergistically enhanced by VISTA/PD-1 double knockout. In addition, VISTA single knockout mice exhibit resistance towards transplanted GL261 glioma [154, 157, 158]. Interestingly, compared to CTLA-4 knockout mice, VISTA knockout mice exhibit no signs for severe autoimmunity pointing out, that other inhibitory receptors compensate for loss of VISTA [157]. The role of VISTA in cancer immune evasion has been demonstrated in melanoma mouse models, where anti- VISTA antibody treatment resulted in enhanced effector function of tumor specific T cells and to decreased tumor growth [156].

Preclinical studies with inhibition of VISTA revealed a progression of autoimmune encephalomyelitis [152], whereby graft- versus-host-reaction could be inhibited by VISTA blockade [153]. In murine tumor models (such as fibrosarcoma [152] or melanoma [159]), VISTA blockade could significantly improve clinic-pathological aspects like tumor growth or overall survival rate. Additionally, this was paralleled by enhanced anti-tumor immunity with increased infiltration, proliferation, and effector function of T-cells [156]. Interestingly, the efficiency of the inhibition of VISTA is independent of missing VISTA expression on the tumor cells, and of the presence of high PD-L1 expression [156, 160].

### CD96

CD96 (also known as Tactile (T cell activation, increased late expression)) is beside CD226 one of the ligands of CD155 [161]. The discovery of CD96 upregulation in T cells and NK cells within human tumors led to the the hypothesis that the inhibition of the CD155/CD96 could essentially influence the tumor elimination [162]. In particular, CD96^−/−^ mice show increased NK-cell activity in response to immune challenge and significant resistance to cancer [163, 164]. In addition, further studies could highlight the role of CD96 in acute myeloid leukaemia (AML) as well as in congenital disease like C syndrome or opitz trigonocephaly [165, 166]. Furthermore CD96 plays a key role in chronic viral disease induced by Hepatitis B [167] or HIV-1 [168], where investigations could reveal that CD96 expression is pathogenetically linked to disease progression [168].

## Clinical trials exploiting reinvigoration of T cells

Although checkpoint inhibition is relatively new, it has become a very attractive single therapy option or a combination partner with other standard care of treatment options. This chapter will summarize in a clear and concise manner recently published clinical trials dealing with checkpoint inhibition (for detailed information see Table 2). To do so, we will concentrate on efficacy and tolerability of the checkpoint inhibitors for CTLA-4, PD-1 and, PD-L1 (Fig. 1), due to the fact that there is too little or even no information about other immune checkpoints in clinical trials at the moment. To anticipate efficacy and possible immune related adverse effects (irAEs), it is important to consider which immune cells and T cell subsets are targeted by the respective therapeutic antibodies. As described in the previous chapters, expression of IRs are not solely restricted to exhausted CD8^+^ Tcells but may also be expressed on T helper, Treg or antigen presenting cells which could amplify or impede therapeutic effects. Hence, CTLA-4 and PD-1/PD-L1 specific antibodies differ in their mode of action. Whereas CTLA-4 antibodies lower the threshold for T cell activation (also of low affine tumor specific naive T cells), antibodies targeting the PD-1/PD-L axis aim at regulating effector T cell activity [42, 169]. In that sense, PD-1/PD-L antibodies do not merely target cytotoxic CD8^+^ T cell subsets but can impede tumor specific Tregs, thereby potentiating tumor specific cytolytic attacks [169]. Monoclonal antibodies that pharmaceutically inhibit CTLA-4 are ipilimumab and tremelimumab. Used as a single therapy, ipilimumab has mostly been investigated in the setting of malignant melanoma and non Hodgkin lymphomas (NHL). In 2015 Eggermont et al. stated in a phase III clinical trial when ipilimumab is given in an adjuvant manner in previously resected stage III melanoma, it significantly improved recurrence-free survival compared with placebo [170]. In combination with glycoprotein 100 (gp100) vaccination or with radiotherapy, ipilimumab improved overall survival or increased the duration of irradiated tumor response [171–173]. Moreover, in combination with the immunostimulator sargramostim, ipilimumab showed longer overall survival in the same setting [174]. Beashey et al. who treated patients suffering from aggressive NHL with ipilimumab after allogenic hematopoetic cell transplantation recorded antitumor responses as well [175]. Nevertheless, a phase II clinical trial in 2015revealed only little clinical activity for ipilimumab when given adjuvant after resection of advanced uveal melanoma [176].

**Table 2:** Clinical trials for checkpoint inhibitors alone and compared to standard care of treatment

Agent (inhibited checkpoint)	Setting	Phase	Treatment	Tumor response	OS (PFS) in MO	Toxicity (irAE grade ≥3)	Ref
Ipilimumab (CTLA-4)	Advanced uveal melanoma	II	Ipilimumap	SD 47%	6.8 (2.8)	Colitis, diarrhea, elevated liver enzymes	[176]
	After complete resection of advanced melanoma	III	Ipilimumab or placebo after complete resection	NM	(26.7 vs 17.1)	Diarrhea, colitis,rash, pruritus, hypo-physitis, elevated liver enzymes	[170]
	Advanced melanoma	II	Ipilimumap	CR 0% PR 10% SD 10% PD 65%	8.7 (2.7)	Elevated liver enzymes	[205]
	Relapse of malignancy after allogeneic hematopoietic stemcell transplan-tation	I	Ipilimumab	ORR 6.9% CR 6.9% PR 3.4%	24.7	Arthritis, pneumonitis	[175]
	Relapsed and refractory B-cell NHL	I	Ipilimumap	NM	NM	Diarrhea, fatigue,	[206]
Treme-limumap (CTLA-4)	Advanced melanoma	III	Tremeli-mumab vs. standard-of-care chemotherapy	NM	12.6 vs 10.7 (at 6 MO 20.3%vs 18.1%)	Diarrhea, colitis, pruritus, rash	[183]
	Advanced melanoma	I	Anti-CD40 + Tremeli-mumab	NM	26.1 (2.5)	Diarrhea, colitis, pruritus, rash	[212]
	Advanced gastric and esophageal adeno-carcinoma	II	Tremeli-mumap	PR 5.6% SD 22%	4.8 (2.8)	Diarrhea, atrial fibrillation, increased liver enzymes	[177]
	Advanced (metastatic) colorectal carcinoma	II	Tremeli-mumap	PR 2.2% PD 95.6%	At 1a 4.8 vs 10.7% (at 6 MO 2.3 vs 2.1%)	Diarrhea, fatigue, colitis	[185]
	Advanced NSCLC	II	Tremeli-mumap vs. best supportive care	PR 4.8% SD 16.6%	20.9% (34%) at 3 MO	Diarrhea, colitis	[213]
	HHC and chronic hepatitis C	II	Tremeli-mumap	SD 58.8% PR 17.6%	8.2 (6.5)	Skin rash, diarrhea, syncope, diverticulitis, depression	[179]
	Advanced malignant mesothelioma	II	Tremeli-mumap	PR 3% SD 38%	11.3	Gastrointes-tinal events, dermatologi-cal events, fever	[214]
Nivolumab (PD-1)	Advanced refractory squamous NSCLC	II	Nivolumab 3 mg/kg every 2 weeks until progression	PR 14.5% SD 26% PD 44%	8.2 (1.9); 1a 40.1%	Fatigue, diarrhea, rash pruritus	[196]
	Untreated melanoma (BRAF wild type vs mutated)	I	Nivolumab + Ipilimumab vs Ipilimumab + placebo	WT [BRAF+] ORR 61% vs 11% [3% vs 1%] CR 16% vs 0% [5% vs 0%] PR 28% vs 4% [7% vs 1%] SD 9% vs 13% [5% vs 7%]	NM	Diarrhea rash. fatigue pruritus, elevated liver enzymes	[187]
	Untreated melanoma without BRAF mutation	III	Nivolumab vs Dacarbazine	ORR 40,0% vs 13,9%	72.9% vs 42.1% at 1a (5.1 vs 2.2)	Fatigue, pruritus, nausea, diarrhea	[186]
	Advanced Squamous-Cell NSCLC	III	Nivolumab vs Docetaxel	ORR 20 vs 9% CR 1 vs 0% PR 26 vs 12% SD 39 vs 47% PD 56% vs 48%	9.2 vs 6.0 (3.5 vs 2.8)	Fatigue, leukopenia	[191]
	Advanced non-Squamous-Cell NSCLC	III	Nivolumab vs Docetaxel	ORR 19% vs 12% CR 4 vs 1% PR 52% vs 35% SD 12;7% vs 21% PD 22.2% vs 14.6%	12.2 vs 9.4 (2.3 vs 4.2)	Fatigue, nausea, diarrhea	[192]
	Relapsed or refractory Hodgkin 's lymphoma	I	Nivolumab	CR 17% PR 70% SD 13%	NM	Leukopenia, stomatitis increased lipase levels, pancreatitis	[206]
	Pretreated advanced NSCLC (s and ns)	I	Nivolumab	ORR 17.1% (16.7% s vs 17.6% ns)	9.9	Rash, Colitis	[190]
	Untreated melanoma	III	Nivolumab vs Nivolumab + Ipilimumab vs Ipilimumab	ORR 14.6% vs 19.2% vs 6.3% CR 8.9% vs 11.5% vs 2.2% PR 34.8% vs 46.2% vs 16.8% SD 10.8% vs 13.1% vs 21.9% PD 37.7% vs 22.6% vs 48.9%	11.5 vs 2.9 vs 6.9	Diarrhea, fatigue, pruritus, rash	[188]
	Platinum resistant ovarian cancer	II	Ipilimumab	CR 10% PR 5% SD 30% PD 50%	20 (3.5)	Lympho-cytopenia, anemia	[215]
	Advanced melanoma after anti CTLA-4 treatment	III	Nivolumab vs investigators choice of chemo	ORR 31.7% vs 10.6% CR 3.3% vs 0% PR 28.3% vs 10.6% SD 23.3% vs 34% PD 35% vs 31.9%	(4.7 vs 4.2)	Anemia, fatigue, vomitting	[189]
	Advanced renal cell carcinoma	III	Nivolumab vs Everolimus	ORR 25% vs 5% CR 1% vs <1%	25.0 vs 19.6 (4.6 vs 4.4)	Fatigue, diarrhea, rash	[216]
Pembroli-zumab (PD-1)	Advanced NSCLC	I	Pembroli-zumab	ORR 19.4%	12.0 (3.7)	Fatigue, rash, diarrhea	[217]
	Advanced triple negative breast cancer	Ib	Pembroli-zumab	ORR 18.5% CR 3.7%; PR 14.8% SD 25.9% PD 48.1%	NM	Anemia, headache,	[218]
	Previously treated advanced non-small-cell lung cancer	II/III	Pembroli-zumab vs Docetaxel	NM	10.4 vs 12.7 vs 8.5 (3.9 vs 4.0 vs 4.0)	Anemia, headache,	[193]
	Advanced melanoma	I	Pembroli-zumab	ORR 38.6% vs 28.6%	23 (4)	Anemia, headache,	[194]
	Progressive metastatic carcinoma with or without mismatch repair-deficiency	II	Pembroli-zumab	ORR 40% vs 78% for mismatch repair-deficienct CRC and 0% vs 11% mismatch repair-proficient colorectal cancer	NM	Lympho-penia, anemia, diarrhea, bowel obstruction, elevated liver enzymes	[195]
	Advanced melanoma	III	Pembrolizumab vs Ipilimumab	ORR 89.4% vs 96.7% vs 87.9%	At 1a 74.1% vs 68.4% (at 6 MO 47.3%vs 46.4% vs 26.5%)	Lympho-penia, anemia, diarrhea, bowel obstruction, elevated liver enzymes	[219]
Atezoli-zumab (PD-L1)	Previously treated metastatic uorthelial carcinoma	II	Atezoli-zumab	ORR 15% CR 5% PR 10% SD 19% PD 51%	NM	Fatigue, decreased appetite, dyspnoea, anemia, colitis	[202]
	Previously treated NSCLC	II	Atezo-lizumab vs Docetaxel	NM	12.6 vs 9.7	Diarrhea, asthenia, neutropenia	[201]

*Abbreviations*: *CR* complete response, *HCC* hepatocellular carcinoma, *irAE* immune related adverse effects, *MO* months, *NM* not mentioned, *NSCLC* non small cell lung cancer, *ORR* overall response rate, *OS* overall survival, *PD* progressive disease, *PFS* progression free survival, *PR* partial response, *SD* stable disease

Tremelimumab as well has been investigated not only in the setting of advanced malignant melanoma, but also in a number of other malignancies like advanced adenocarcinomas of the gastrointestinal tract, non small cell lung carcinoma (NSCLC) and hepatocellular carcinoma (HCC) as well as malignant mesothelioma [177–182]. Concerning malignant melanoma, in 2013 Ribas et al. were not able to demonstrate a statistically significant survival advantage for tremelimumab compared to standard-of-care chemotherapy in patients suffering from advanced melanoma [183]. But in combination with high dose interferon-α treatment of malignant melanomas showed significant therapeutic benefit [184]. The clinical phase II studies dealing with adenocarcinomas of the esophagus and the colon showed disappointing response rates, not supporting further investigations [177, 185]. In contrast, tremelimumab showed antitumor and antiviral effects in patients suffering from HCC on the basis of hepatitis C-virus infections [179].

The PD-1 inhibiting agents, Nivolumab and Pembrolizumab, were also used in clinical trials to treat malignant melanoma. In a phase III clinical trial, performed by Robert et al., nivolumab showed significant improvements in overall survival and progression free survival compared with dacarbazine. This trial setting focused on untreated melanoma without BRAF mutation [186]. Additionally, Postow et al. and others demonstrated that the combination of nivolumab and ipilimumab had significant advantages over single nivolumab therapy or placebo alone concerning progression-free survival [187, 188]. Even as a second line therapy nivolumab seems to improve outcome in malignant melanoma. In this phase III trial, ipilumumab pretreated advanced melanoma patients were either treated with nivolumab or investigators choice of chemotherapy. In this setting nivolumab demonstrated higher objective response rates than the alternative available chemotherapy [189]. In the setting of squamous or non squamous NSCLC, nivolumab seems to improve survival rates in previously heavily treated patients [190]. It even showed a better performance compared to docetaxel [191, 192]. Similar to that, pembrolizumab prolonged overall survival compared to docetaxel in NSCLC in a phase II/III clinical trial [193]. Obviously, patients with malignant melanoma were treated with pembrolizumab in a clinical trial as well. Ribas et al. were able to show that pembrolizumab prolonged progression-free survival and overall survival compared to ipilimumab. In another phase I clinical trial pembrolizumab improved objective response and survival rates [194]. In addition, Le et al. showed another very interesting feature of pembrolizumab. They performed a phase II clinical trial in which they were able to investigate that mismatch-repair deficiency predicted clinical effect of pembrolizumab in patients suffering from colorectal carcinoma [195], implying that response rates and clinical benefit from anti-PD1 therapies is correlating with high non-synonymous mutation load, which associates with the presence of tumor associated neoantigens [195, 196]. It was suggested that there is a general correlation of mutation load within tumor DNA and efficacy of immune checkpoint inhibition, irrespective of targeting PD-1 or its ligand, likely by an increased expression of tumor associated neoantigens [195–197]. While tumors with deficiencies in DNA mismatch-repair were found to have a better response toPD-1 blockade [195], it will certainly be clinically relevant to assess other surrogate markers which predict response to immune checkpoint blockade. These markers could likely be mutations in other DNA repair genes but also expression levels of DNA-mutating enzymes, such as family members of the AID/APOBEC deaminases, which could lead to increased mutation load in tumor DNA [198]. In addition, a similar correlation of treatment response and mutation load has been shown for melanoma patients treated with CTLA-4 [194, 195].

Pidilizumab, another PD-1 inhibitor, was used in a combination therapy in two different phase II clinical studies. Relapsed follicular lymphoma patients treated with pidilizumab in combination with rituximab exhibited an overall response rate of 66% and a complete response rate of 52% [199]. In the setting of diffuse large B cell lymphoma, patients treated with pidilizumab after hematopoietic stem cell transplantation showed an overall response rate of 51% and complete response in 34%, although 37% of patients showed a progressive disease in the same clinical trial [200].

Unlike PD-1 targeting antibodies, the PD-L1 specific antibody atezolizumab is not primarily used in the setting of melanoma. In previously treated NSCLC patients, atezolizumab improved survival compared with docetaxel in correlation with PD-L1 expression in the tumor and in tumor infiltrating immune cells [201]. Similar effects on survival were seen in another study dealing with previously metastatic urothelial carcinoma [202]. In combination with cobimetinib, a selective mitogen activated protein kinase (MAP2K1) inhibitor, atezolizumab ameliorated response rates even in mismatch repair proficient metastatic colorectal cancer [203].

Regarding the immune related adverse events of checkpoint inhibitors, all mentioned antibodies show similar immune related adverse events (irAEs, see Tables 2 and 3). Adverse events of grade 3 or higher affected most of the gastrointestinal tract, the skin, the liver function and the hematopoietic system (for more details see Tables 2 and 3). Diarrhea or colitis was observed in almost all clinical trials. However, the majority of adverse events were acceptable and mostly easy to manage [204–206]. Compared to standard chemotherapy, some investigators stated a much better tolerability for checkpoint inhibitors [189, 192, 201]. Moreover, a combination of checkpoint inhibition with ipilimumab and radiotherapy did not show an increase in adverse events [172]. Furthermore, clinical trials investigating combination therapies with standard of care therapies like exemestane in breast cancer, bicalutamide in prostate cancer, rituximab in follicular lymphoma or gemcitabine in pancreatic cancer, showed usually a satisfactory adverse events profile [199, 207–209]).

**Table 3:** Clinical trials for checkpoint inhibitors in combination with standard care of treatment

Agent (inhibited check-point)	Setting	Phase	Treatment	Tumor response	OS (PFS) in months	Toxicity irAE grade ≥3	Ref.
Ipilimumab (CTLA-4)	Advanced melanoma	III	Ipilimumab or Ipilimumab + glycoprotein 100 or glycoprotein 100 only	NM	10 vs 10.1 vs 6.4 (2.76 vs 2.86 vs 2.76)	Diarrhea, nausea, constipation, vomiting, abdominal pain	[171]
	Advanced melanoma	Retrospective	Ipilimumab or maintenance + median 30 Gy	NM	9 vs 39	NM	[172]
	Advanced melanoma	Retrospective	Ipilimumab vs Ipilimumab + radiotherapy	NM	10.2 vs 19.6	Rash, colitis, GI, fatigue	[173]
	Advanced melanoma	I	Ipilimumab plus radiotherapy	NM	10.7 (3.8)	Anemia, diarrhea, colitis	[220]
	Metastatic melanoma	II	Ipilimumab + sargramostim vs Ipilimumab alone	NM	17.5 vs 12.7 (3.1 vs 3.1)	Diarrhea, rash, colitis, elevated liver enzymes	[174]
	Metastatic NSCLC	I	Ipilimumab + Paclitaxel vs Ipilimumab + Carboplatin	NM	NM	Adrenal insuffiency, enterocolitis	[221]
	Advanced, bone metastasis, castration-resistant prostate cancer	III	Ipilimumab or placebo after 8 GY	NM	11.2 vs 10.2 (4.0 vs 3.1; at 6 MO 30.7% vs 18.1%)	Diarrhea, colitis	[222]
Tremel-imumap (CTLA-4)	Prostate cancer (PSA-recurrent)	I	Tremeli-mumab + Bicalutamide	NM	NM	Colitis	[208]
	Advanced breast cancer	I	Tremeli-mumab + Exemestane	SD 42%	NM	Diarrhea, rash	[207]
	Metastatic pancreatic cancer	I	Tremeli-mumab + Gemcitabine	PR 10.5%	7.4	Asthenia, nausea, diarrhea	[223]
	Advanced melanoma (or solid tumors)	I	Tremeli-mumab + PF-3512676 (CPG 7909) = Toll like receptor 9 inhibitor	NM	19	Diarrhea, hypophy-sitis, colitis, nausea, vomiting, pruritus, rash, neutropenia, rectal Bleeding	[224]
	Advanced melanoma	II	Trimilimumab + high dose INFalpha (HDI)	ORR 24% CR 11% PR 14% SD 38%	21 (6.4)	Diarrhea, colitis, elevated liver enzymes, rash, fatigue, anxiety/depression	[184]
	Metastatic renal cell carcinoma	I	Tremeli-mumab + sunitinib	PR 42.8%; SD 9.5%	2.8–18.2MO	Fatigue, mucositis, dypnea	[225]
Nivolumab (PD-1)	Resected advanced melanoma	II	Adjuvant Nivolumab + multi-peptide vaccine (gp100, MART-1 & NY-ESO-1 with Montanide ISA 51 VG)	NM	At 1a 87% At 2a 82%	Colitis, enteritis, rash, hypokalemia	[226]
Pidilizumab (PD-1)	Relapsed follicular lymphoma	II	Pidilizumab + Rituximab	ORR 66% CR 52% PR 14%	NM	No grade 3 or higher irAE	[199]
	DLBCL	II	Pidilizumab after autologous hematopoietic stem- cell transplan-tation	ORR 51% CR 34% PR 17% SD 37% PD 11%	At 16 MO 0.85% (at 16 MO 0,72%)	Thrombo-cytopenia, anemia, pyrexia, renal failure,	[200]
Atezoli-zumab (PD-L1)	Microsatellite stable metastatic colorectal cancer	Ib	Combination of cobimetinib and ateolizumab	ORR 17% and 20% in KRAS-mutant tumors	At 6 MO 72%	NM	[203]

*Abbreviations*: *CR* complete response, *irAE* immune related adverse effects, *MO* months, *NM* not mentioned, *NSCLC* non small cell lung cancer, *ORR* overall response rate, *OS* overall survival, *PD* progressive disease, *PFS* progression free survival, *PR* partial response, *SD* stable disease

## Conclusions

The results of numerous clinical trials using immune checkpoint inhibitors are very encouraging. Blocking antibodies for CTLA-4, PD-1 or PD-L1 seem to have a strong therapeutic potential when given alone or in combination with standard care of treatment in many different tumor entities. Additionally, checkpoint inhibitors adverse events profiles do not seem to be much worse than profiles of standard chemotherapies, but due to the fact that recently published clinical trials were in phase I or II, these encouraging data needs to be verified in more phase III clinical trials with longer follow up and larger numbers of patients. In addition, future challenges will be to elucidate proper pretreatments or combination therapies to increase clinical benefit of checkpoint inhibition also in cancer with initial low non-synonymous mutation load or low neoantigen expression.

## Abbreviations

AKT: proteinkinase B
BTLA: B and T lymphocyte attenuator
CR: complete response
CTLA-4: cytotoxic T-lymphocyte-associated protein 4
EAE: experimental autoimmune encephalomyelitis
Eomes: eomesodermin
Gal-9: galectin-9
HBV: hepatitis B virus
HCC: hepatocellular carcinoma
HCC: hepatocellular carcinoma
HCV: hepatitis C virus
HIV: human immunodeficiency virus
HTLV1: human T lymphotropic virus 1
HVEM: herpesvirus entry mediator
IgSV: immunoglobulin superfamily
IR: inhibitory receptor
irAE: immune related adverse effects
ITAM: immunoreceptor tyrosine-based activation motif
ITIM: immunoreceptor tyrosine- based inhibitory motif
ITSM: immunoreceptor tyrosine- based switch motif
ITT: immunoglobulin tail tyrosine
LAG-3: lymphocyte-activated gene-3
LCMV: lymphocytic choriomeninigits virus
MO: months
NHL: non Hodgkin lymphoma
NK: natural killer cell
NKT: natural killer T cell
NM: not mentioned
NOD: non-obese diabetic
NSCLC: non small cell lung cancer
NSCLC: non-small cell lung cancer
ORR: overall response rate
OS: overall survival
PD: progressive disease
PD-1: programmed cell death 1
PD-1H: PD-1 homolog
PD-L1: programmed cell death-ligand 1
PD-L2: programmed cell death-ligand 1
PFS: progression free survival
PI3K: phosphatidylinositide 3-kinases
PR: partial response
PVR: poliovirus receptors
SD: stable disease
SIV: simian immunodeficiency virus
SLAM: signaling lymphocyte activation molecule
T-bet: T-box transcription factor TBX21
TCR: T cell receptor
T_FH_: follicular helper T cells
TIGIT: T cell immunoreceptor with Ig and ITIM domains
TILs: tumor-infiltrating T cell
TIM-3: T-cell immunoglobulin and mucin- containing protein 3
TLR: toll-like receptor
Tr1: type 1 regulatory T cells
Treg: regulatory T cells
Tregs: regulatory T cells
VISTA: V domain Ig suppressor of T cells activation
